# Assisted reproductive technology in Japan: A summary report for 2018 by the Ethics Committee of the Japan Society of Obstetrics and Gynecology

**DOI:** 10.1002/rmb2.12358

**Published:** 2020-11-20

**Authors:** Osamu Ishihara, Seung Chik Jwa, Akira Kuwahara, Yukiko Katagiri, Yoshimitsu Kuwabara, Toshio Hamatani, Miyuki Harada, Yutaka Osuga

**Affiliations:** ^1^ Department of Obstetrics and Gynecology Saitama Medical University Saitama Japan; ^2^ Department of Obstetrics and Gynecology Graduate School of Biomedical Sciences Tokushima University Tokushima Japan; ^3^ Department of Obstetrics and Gynecology Faculty of Medicine Toho University Tokyo Japan; ^4^ Department of Obstetrics and Gynecology Nippon Medical School Tokyo Japan; ^5^ Department of Obstetrics and Gynecology School of Medicine Keio University Tokyo Japan; ^6^ Department of Obstetrics and Gynecology Faculty of Medicine The University of Tokyo Tokyo Japan

**Keywords:** ART registry, freeze‐all strategy, in vitro fertilization, intracytoplasmic sperm injection, Japan Society of Obstetrics and Gynecology

## Abstract

**Purpose:**

Since 2007, the Japan Society of Obstetrics and Gynecology (JSOG) has collected cycle‐based data for assisted reproductive technology (ART) in an online registry. Here, we present the characteristics and treatment outcomes of ART cycles registered during 2018.

**Methods:**

The Japanese ART registry consists of cycle‐specific information for all ART treatment cycles implemented at 621 participating facilities. We conducted descriptive analyses for such cycles registered for 2018.

**Results:**

In total, 454 893 treatment cycles and 56 979 neonates were reported in 2018: both increased from 2017. The mean maternal age was 38.0 years (standard deviation ± 4.7). Of 247 402 oocyte retrievals, 118 378 (47.8%) involved freeze‐all‐embryos cycles; fresh embryo transfer (ET) was performed in 50 463 cycles: a decreasing trend since 2015. A total of 199 914 frozen‐thawed ET cycles were reported, resulting in 69 357 pregnancies and 49 360 neonates born. Single ET (SET) was performed in 82.2% of fresh transfers and 83.4% of frozen‐thawed cycles, with singleton pregnancy/live birth rates of 97.2%/97.2% and 97.0%/97.2%, respectively.

**Conclusions:**

Total ART cycles and subsequent live births increased in 2018. SET was performed in over 80% of cases, and the mode of ET has shifted continuously from using fresh embryos to frozen‐thawed ones compared with previous years.

## 1 INTRODUCTION

Since the first baby in Japan conceived as a result of in vitro fertilization (IVF) was born in 1983, the number of assisted reproductive technology (ART) cycles has increased dramatically each year. According to the latest preliminary report from the International Committee Monitoring Assisted Reproductive Technologies for ART worldwide in 2016, Japan was the second largest user of ART worldwide in terms of the annual total number of treatment cycles performed.^1^

Because it is essential to monitor the trend and situations of ART treatments implemented in our country, in 1986 the Japan Society of Obstetrics and Gynecology (JSOG) began an ART registry system and launched an online registration system in 2007. Since then, cycle‐specific information for all ART treatment cycles performed in ART facilities has been collected. The aim of this report is to describe the characteristics and treatment outcomes of registered ART cycles during 2018 in comparison with previous years.^2^

## 2 MATERIALS AND METHODS

Since 2007, the JSOG has requested all participating ART clinics and hospitals to register cycle‐specific information for all ART treatment cycles. The information includes patient characteristics, information on the specific ART treatment, and pregnancy and obstetric outcomes. Detailed information collected in the registry has been reported previously.^3^ For ART cycles performed between January 1, 2018, and December 31, 2018, the JSOG requested registration of the information via an online registry system by the end of November 2019.

Using the registry data for 2018, we performed a descriptive analysis to investigate the characteristics and treatment outcomes of registered cycles. First, the numbers of registered cycles for the initiation of treatment, oocyte retrievals, fresh embryo transfer (ET) cycles, freeze‐all‐embryos/oocytes cycles (henceforth “freeze‐all”), frozen‐thawed embryo transfer (FET) cycles, pregnancies, and neonates were compared with those in previous years. Second, the characteristics of registered cycles and treatment outcomes were described for fresh and FET cycles. Fresh cycles were stratified according to fertilization method including IVF, intracytoplasmic sperm injection (ICSI), gamete intrafallopian transfer (GIFT), and included cycles with oocyte freezing based on medical indications. Treatment outcomes included pregnancy, miscarriage, and live birth rates, multiple pregnancies defined according to the numbers of gestational sacs in utero and of neonates. Pregnancy outcomes included ectopic pregnancy, heterotopic pregnancy, artificially induced abortion, stillbirth, and fetal reduction. Third, the treatment outcomes of pregnancy, live birth, miscarriage, and multiple pregnancy rates were analyzed according to patient age. Last, we also recorded the treatment outcomes for FET cycles using frozen‐thawed oocytes based on medical indications.

## 3 RESULTS

In Japan, there were 622 registered ART facilities in 2018, of which 621 participated in the ART registration system. A total of 592 registered facilities actually implemented some form of ART treatment in 2018 while 29 did not. Trends in the numbers of registered cycles, oocyte retrievals, pregnancies, and neonates born as a result of IVF, ICSI, and FET cycles from 1985 to 2018 are shown in Table 1. In 2018, 454 893 cycles were registered and 56 979 neonates were recorded. The total number of registered cycles for fresh IVF and ICSI followed by ET had decreased from the previous year in 2017. However, in 2018, the registered cycles and oocyte retrieval cycles increased from 2017 both for IVF and ICSI. Among registered fresh ET cycles, 63.2% were ICSI. The numbers of freeze‐all cycles increased both in IVF and ICSI cycles, so the numbers of neonates born after fresh ET cycles have been decreasing because of fewer such treatments. In contrast, the number of FET cycles increased continuously; in 2018, there were 203 482 (a 2.2% increase from 2017), resulting in 69 395 pregnancies and 49 383 neonates.

**TABLE 1:** Trends in numbers of registered cycles, oocyte retrievals, pregnancies and neonates according to IVF, ICSI, and frozen‐thawed embryo transfer cycles, Japan, 1985‐2018

Year	Fresh cycles												FET cycles^c^			
	IVF^a^						ICSI^b^									
	No. of registered cycles	No. of egg retrieval	No. of freeze‐all cycles	No. of ET cycles	No. of cycles with pregnancy	No. of neonates	No. of registered cycles	No. of oocyte retrievals	No. of freeze‐all cycles	No. of ET cycles	No. of cycles with pregnancy	No. of neonates	No. of registered cycles	No. of ET cycles	No. of cycles with pregnancy	No. of neonates
1985	1195	1195		862	64	27										
1986	752	752		556	56	16										
1987	1503	1503		1070	135	54										
1988	1702	1702		1665	257	114										
1989	4218	3890		2968	580	446							184	92	7	3
1990	7405	6892		5361	1178	1031							160	153	17	17
1991	11 177	10 581		8473	2015	1661							369	352	57	39
1992	17 404	16 381		12 250	2702	2525	963	936		524	42	35	553	530	79	66
1993	21 287	20 345		15 565	3730	3334	2608	2447		1271	176	149	681	597	86	71
1994	25 157	24 033		18 690	4069	3734	5510	5339		4114	759	698	1303	1112	179	144
1995	26 648	24 694		18 905	4246	3810	9820	9054		7722	1732	1579	1682	1426	323	298
1996	27 338	26 385		21 492	4818	4436	13 438	13 044		11 269	2799	2588	2900	2676	449	386
1997	32 247	30 733		24 768	5730	5060	16 573	16 376		14 275	3495	3249	5208	4958	1086	902
1998	34 929	33 670		27 436	6255	5851	18 657	18 266		15 505	3952	3701	8132	7643	1748	1567
1999	36 085	34 290		27 455	6812	5870	22 984	22 350		18 592	4702	4247	9950	9093	2198	1812
2000	31 334	29 907		24 447	6328	5447	26 712	25 794		21 067	5240	4582	11 653	10 719	2660	2245
2001	32 676	31 051		25 143	6749	5829	30 369	29 309		23 058	5924	4862	13 034	11 888	3080	2467
2002	34 953	33 849		26 854	7767	6443	34 824	33 823		25 866	6775	5486	15 887	14 759	4094	3299
2003	38 575	36 480		28 214	8336	6608	38 871	36 663		27 895	7506	5994	24 459	19 641	6205	4798
2004	41 619	39 656		29 090	8542	6709	44 698	43 628		29 946	7768	5921	30 287	24 422	7606	5538
2005	42 822	40 471		29 337	8893	6706	47 579	45 388		30 983	8019	5864	35 069	28 743	9396	6542
2006	44 778	42 248		29 440	8509	6256	52 539	49 854		32 509	7904	5401	42 171	35 804	11 798	7930
2007	53 873	52 165	7626	28 228	7416	5144	61 813	60 294	11 541	34 032	7784	5194	45 478	43 589	13 965	9257
2008	59 148	57 217	10 139	29 124	6897	4664	71 350	69 864	15 390	34 425	7017	4615	60 115	57 846	18 597	12 425
2009	63 083	60 754	11 800	28 559	6891	5046	76 790	75 340	19 046	35 167	7330	5180	73 927	71 367	23 216	16 454
2010	67 714	64 966	13 843	27 905	6556	4657	90 677	88 822	24 379	37 172	7699	5277	83 770	81 300	27 382	19 011
2011	71 422	68 651	16 202	27 284	6341	4546	102 473	100 518	30 773	38 098	7601	5415	95 764	92 782	31 721	22 465
2012	82 108	79 434	20 627	29 693	6703	4740	125 229	122 962	41 943	40 829	7947	5498	119 089	116 176	39 106	27 715
2013	89 950	87 104	25 085	30 164	6817	4776	134 871	134 871	49 316	41 150	8027	5630	141 335	138 249	45 392	32 148
2014	92 269	89 397	27 624	30 414	6970	5025	144 247	141 888	55 851	41 437	8122	5702	157 229	153 977	51 458	36 595
2015	93 614	91 079	30 498	28 858	6478	4629	155 797	153 639	63 660	41 396	8169	5761	174 740	171 495	56 888	40 611
2016	94 566	92 185	34 188	26 182	5903	4266	161 262	159 214	70 387	38 315	7324	5166	191 962	188 338	62 749	44 678
2017	91 516	89 447	36 441	22 423	5182	3731	157 709	155 758	74 200	33 297	6757	4826	198 985	195 559	67 255	48 060
2018	92 552	90 376	38 882	20 894	4755	3402	158 859	157 026	79 496	29 569	5886	4194	203 482	200 050	69 395	49 383

Abbreviations: ET, embryo transfer; FET, Frozen‐thawed embryo transfer; ICSI, intracytoplasmic sperm injection; IVF, in vitro fertilization.

^a^ Including gamete intrafallopian transfer.

^b^ Including split‐ICSI cycles.

^c^ Including cycles using frozen‐thawed oocytes.

**FIGURE 1:**
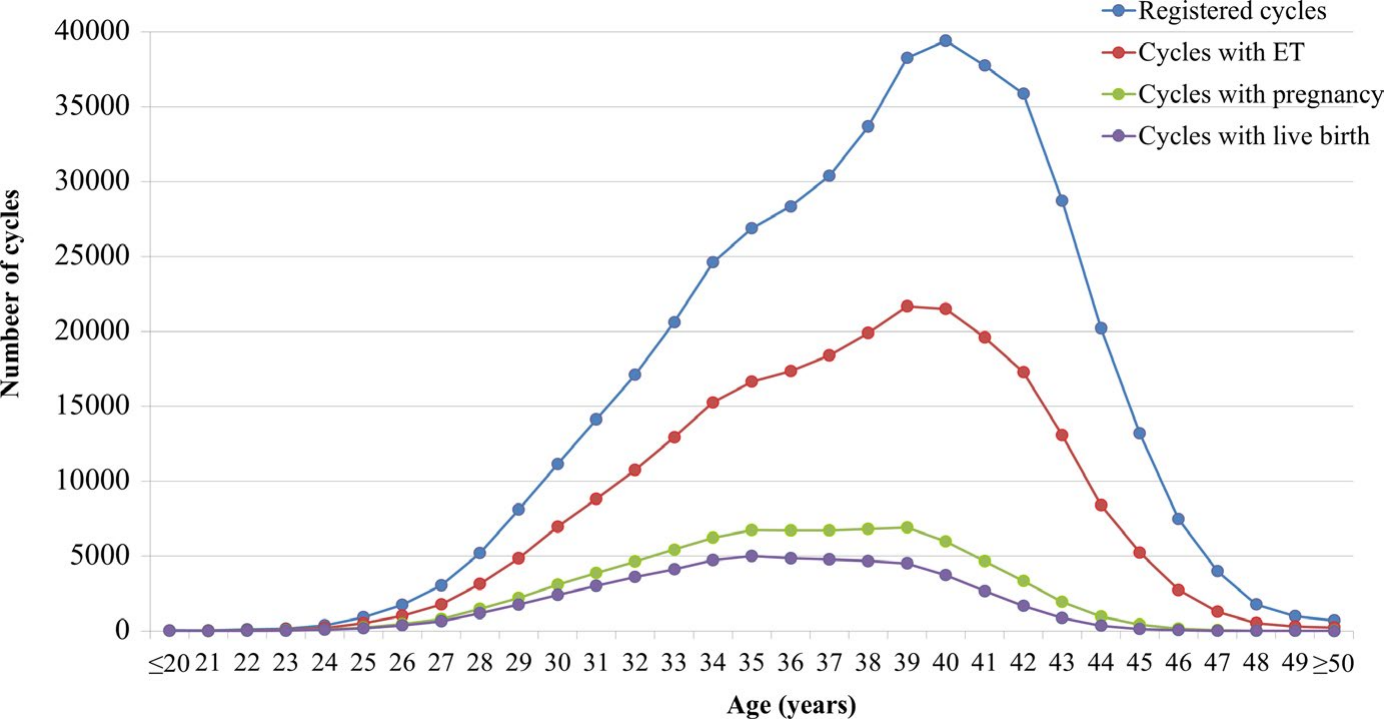
Age distributions of all registered cycles, different subgroups of cycles for ET, pregnancy, and live birth in 2018. Adapted from the Japan Society of Obstetrics and Gynecology ART Databook 2018 (http://plaza.umin.ac.jp/~jsog‐art/2018data_20201001.pdf). ET, embryo transfer

The distributions of patient age in registered cycles and different subgroups of cycles with ET, pregnancy, and live births are shown in Figure 1. The mean patient age for registered cycles was 38.0 years (standard deviation [SD] ±4.7), and 41.8% of registered cycles were for women in their 40s; the mean age for pregnancy and live birth cycles was 36.0 years (SD ± 4.1) and 35.6 years (SD ± 4.0), respectively.

The detailed characteristics and treatment outcomes of registered fresh cycles are shown in Table 2. There were 88 072 registered IVF cycles, 28 546 split‐ICSI cycles, 127 974 ICSI cycles using ejaculated spermatozoa, 2339 ICSI cycles using testicular sperm extraction (TESE), 25 GIFT cycles, 644 cycles for oocyte freezing based on medical indications, and 3811 other cycles. Of the 247 402 cycles with oocyte retrieval, 118 378 (47.8%) were freeze‐all cycles. The pregnancy rate per ET was 22.8% for IVF and 18.7% for ICSI using ejaculated spermatozoa. Single ET was performed at a rate of 82.2%, with a pregnancy rate of 21.4%. Live birth rates per ET were 15.9% for IVF, 12.7% for ICSI using ejaculated spermatozoa, and 11.3% for ICSI with TESE. The singleton pregnancy rate and live birth rate were 97.2% and 97.2%, respectively.

**TABLE 2:** Characteristics and treatment outcomes of registered fresh ART cycles in assisted reproductive technology, Japan, 2018

Variables	IVF–ET	Split	ICSI		GIFT	Frozen oocyte	Others^a^	Total
			Ejaculated sperm	TESE				
No. of registered cycles	88 072	28 546	127 974	2339	25	644	3811	251 411
No. of egg retrieval	86 021	28 267	126 422	2337	25	638	3692	247 402
No. of fresh ET cycles	20 403	5058	23 981	530	25	0	466	50 463
No. of freeze‐all‐embryos	37 116	19 728	58 454	1314	0	538	1228	118 378
No. of cycles with pregnancy	4648	1310	4487	89	4	0	103	10 641
Pregnancy rate per ET	22.8%	25.9%	18.7%	16.8%	16.0%	‐	22.1%	21.1%
Pregnancy rate per egg retrieval	5.4%	4.6%	3.6%	3.8%	16.0%	‐	2.8%	4.3%
Pregnancy rate per egg retrieval excluding freeze‐all‐embryos	9.5%	15.3%	6.6%	8.7%	16.0%	‐	4.2%	8.3%
SET cycles	17 014	4449	19 292	340	2	‐	402	41 499
Pregnancy following SET cycles	3933	1176	3624	58	0	‐	92	8883
Rate of SET cycles	83.4%	88.0%	80.5%	64.2%	8.0%	‐	86.3%	82.2%
Pregnancy rate following SET cycles	23.1%	26.4%	18.8%	17.1%	0.0%	‐	22.9%	21.4%
Miscarriages	1161	273	1229	28	0	‐	20	2711
Miscarriage rate per pregnancy	25.0%	20.8%	27.4%	31.5%	0.0%	‐	19.4%	25.5%
Singleton pregnancies^b^	4408	1256	4247	85	3	‐	98	10 097
Multiple pregnancies^b^	113	29	143	1	0	‐	3	289
Twin pregnancies^b^	109	29	142	1	0	‐	3	284
Triplet pregnancies^b^	4	0	1	0	0	‐	0	5
Quadruplet pregnancies^b^	0	0	0	0	0	‐	0	0
Multiple pregnancy rate^b^	2.5%	2.3%	3.6%	1.2%	0.0%	‐	3.0%	2.8%
Live births	3246	965	3045	60	3	‐	78	7397
Live birth rate per ET	15.9%	19.1%	12.7%	11.3%	12.0%	‐	16.7%	14.7%
Total number of neonates	3319	983	3150	61	3	‐	80	7596
Singleton live births	3164	947	2926	59	3	‐	76	7175
Twin live births	76	18	112	1	0	‐	2	209
Triplet live births	1	0	0	0	0	‐	0	1
Quadruplet live births	0	0	0	0	0	‐	0	0
Pregnancy outcomes						‐		
Ectopic pregnancies	59	20	56	0	1	‐	1	137
Heterotopic pregnancy	0	0	0	0	0	‐	0	0
Artificial abortions	25	5	25	1	0	‐	1	57
Still births	18	4	5	0	0	‐	0	27
Fetal reductions	1	0	0	0	0	‐	0	1
Unknown cycles for pregnancy outcomes	104	32	121	0	0	‐	3	260

Abbreviations: ET, embryo transfer; GIFT, gamete intrafallopian transfer; ICSI, intracytoplasmic sperm injection; IVF–ET; in vitro fertilization‐embryo transfer; SET, single embryo transfer; TESE, testicular sperm extraction.

^a^ Others including zygote intrafallopian transfer (ZIFT).

^b^ Singleton, twin, triplet and quadruplet pregnancies were defined according to the numbers of gestational sacs in utero.

The characteristics and treatment outcomes of FET cycles are shown in Table 3. There were 203 246 registered cycles, among which FET was performed in 199 914, leading to 69 357 pregnancies (pregnancy rate per FET = 34.7%). The miscarriage rate per pregnancy was 25.5%, resulting in a 24.1% live birth rate per ET. Single ET was performed at a rate of 84.4%, and the singleton pregnancy and live birth rates were 97.0% and 97.2%, respectively.

**TABLE 3:** Characteristics and treatment outcomes of FET cycles in ART clinics in Japan for 2018

Variables	FET	Others^a^	Total
No. of registered cycles	202 229	1017	203 246
No. of FET	199 022	892	199 914
No. of cycles with pregnancy	69 072	285	69 357
Pregnancy rate per FET	34.7%	32.0%	34.7%
SET cycles	167 898	743	168 641
Pregnancy following SET cycles	59 899	242	60 141
Rate of SET cycles	84.4%	83.3%	83.4%
Pregnancy rate following SET cycles	35.7%	32.6%	35.7%
Miscarriages	17 601	69	17 670
Miscarriage rate per pregnancy	25.5%	24.2%	25.5%
Singleton pregnancies^b^	65 556	266	65 822
Multiple pregnancies^b^	2015	9	2024
Twin pregnancies^b^	1980	8	1988
Triplet pregnancies^b^	31	1	32
Quadruplet pregnancies^b^	3	0	3
Multiple pregnancy rate^b^	3.0%	3.3%	3.0%
Live births	47 873	208	48 081
Live birth rate per FET	24.1%	23.3%	24.1%
Total number of neonates	49 148	212	49 360
Singleton live births	46 432	200	46 632
Twin live births	1335	6	1341
Triplet live births	14	0	14
Quadruplet live births	1	0	1
Pregnancy outcomes			
Ectopic pregnancies	350	1	351
Heterotopic pregnancy	9	0	9
Artificial abortions	272	0	272
Still births	236	1	237
Fetal reduction	16	0	16
Unknown cycles for pregnancy outcomes	1999	3	2002

Abbreviations: FET, frozen‐thawed embryo transfer; SET, single embryo transfer.

^a^ Including cycles using frozen‐thawed oocyte.

^b^ Singleton, twin, triplet and quadruplet pregnancies were defined according to the numbers of gestational sacs in utero.

**FIGURE 2:**
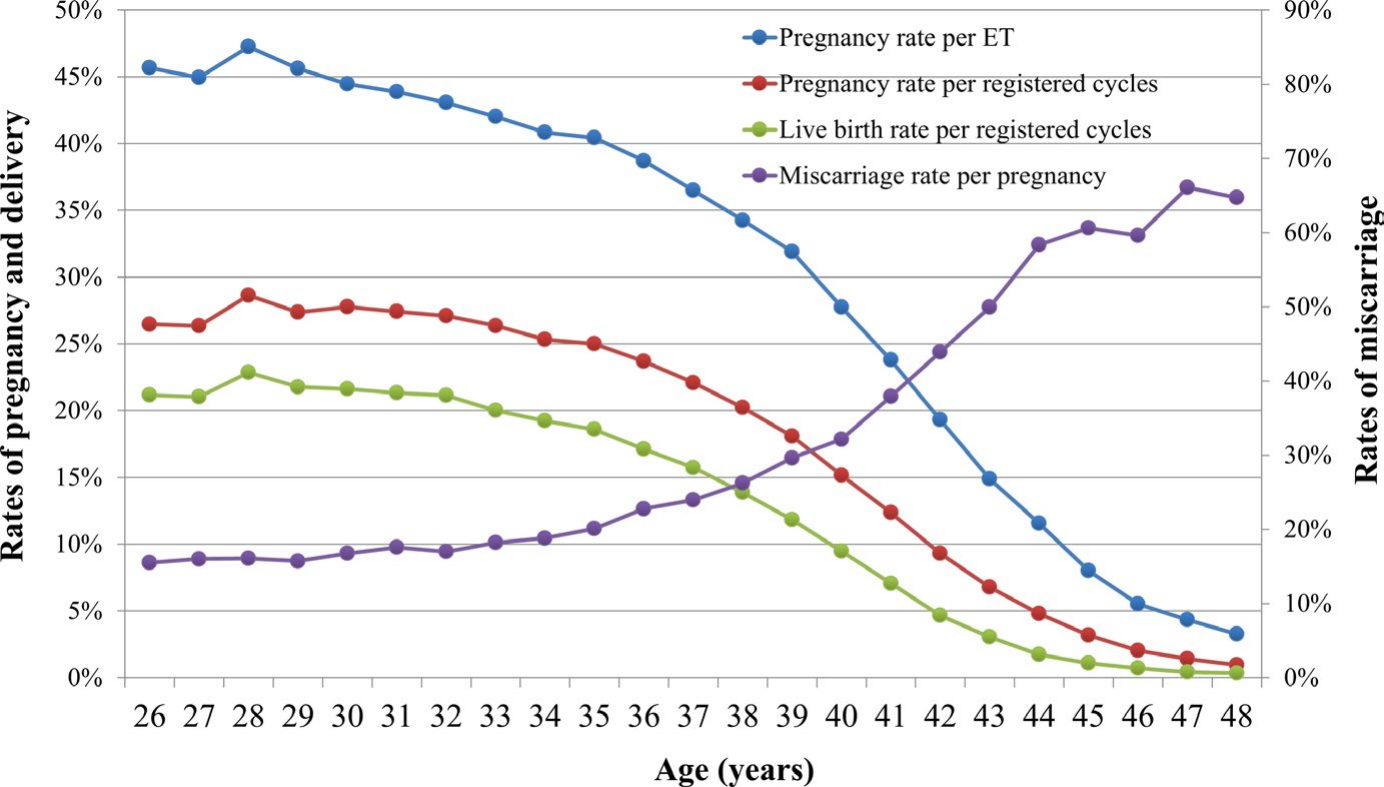
Pregnancy, live birth, and miscarriage rates, according to patient age, among all registered cycles in 2018. Adapted from the Japan Society of Obstetrics and Gynecology ART Databook 2018 (http://plaza.umin.ac.jp/~jsog‐art/2018data_20201001.pdf). ET, embryo transfer

Treatment outcomes of registered cycles, including pregnancy, miscarriage, live birth, and multiple pregnancy rates, according to maternal age, are shown in Table 4. Similarly, the distribution of pregnancy, live birth, and miscarriage rates according to maternal age are shown in Figure 2. The pregnancy rate per ET exceeded 40% up to 35 years of age; this rate gradually fell below 30% after age 40 and below 10% after age 45. The miscarriage rate was below 20% under age 35 years but gradually increased to 32.1% and 49.9% for those aged 40 and 43 years, respectively. The live birth rate per registered cycle was around 20% up to 33 years of age and decreased to 9.5% and 3.1% at ages 40 and 43 years, respectively. Multiple pregnancy rates varied between 2% and 3% across most age groups.

**TABLE 4:** Treatment outcomes of registered cycles according to patient age, Japan, 2018

Age (years)	No. of registered cycles	No. of ET cycles	Pregnancy	Multiple pregnancies^a^	Miscarriage	Live birth	Pregnancy rate per ET	Pregnancy rate per registered cycles	Live birth rate per registered cycles	Miscarriage rate per pregnancy	Multiple pregnancy rate^a^
≤20	49	6	3	0	0	3	50.0%	6.1%	6.1%	0.0%	0.0%
21	29	8	1	0	0	1	12.5%	3.4%	3.4%	0.0%	0.0%
22	103	48	22	2	3	16	45.8%	21.4%	15.5%	13.6%	9.5%
23	155	79	35	2	5	26	44.3%	22.6%	16.8%	14.3%	5.7%
24	384	205	98	1	15	82	47.8%	25.5%	21.4%	15.3%	1.0%
25	926	514	231	0	33	177	44.9%	24.9%	19.1%	14.3%	0.0%
26	1757	1018	465	18	72	372	45.7%	26.5%	21.2%	15.5%	4.0%
27	3037	1780	800	13	128	639	44.9%	26.3%	21.0%	16.0%	1.7%
28	5199	3148	1488	32	239	1188	47.3%	28.6%	22.9%	16.1%	2.2%
29	8098	4859	2216	74	348	1763	45.6%	27.4%	21.8%	15.7%	3.4%
30	11 128	6951	3090	92	518	2407	44.5%	27.8%	21.6%	16.8%	3.0%
31	14 116	8826	3872	100	680	3011	43.9%	27.4%	21.3%	17.6%	2.6%
32	17 101	10 755	4632	117	786	3615	43.1%	27.1%	21.1%	17.0%	2.6%
33	20 594	12 921	5428	135	987	4122	42.0%	26.4%	20.0%	18.2%	2.6%
34	24 600	15 257	6229	176	1171	4732	40.8%	25.3%	19.2%	18.8%	2.9%
35	26 892	16 639	6727	211	1350	5002	40.4%	25.0%	18.6%	20.1%	3.2%
36	28 339	17 346	6714	252	1528	4848	38.7%	23.7%	17.1%	22.8%	3.8%
37	30 377	18 393	6711	197	1606	4778	36.5%	22.1%	15.7%	23.9%	3.0%
38	33 679	19 893	6813	217	1787	4670	34.2%	20.2%	13.9%	26.2%	3.3%
39	38 256	21 684	6917	203	2049	4521	31.9%	18.1%	11.8%	29.6%	3.0%
40	39 410	21 510	5969	162	1918	3733	27.7%	15.1%	9.5%	32.1%	2.8%
41	37 736	19 608	4664	126	1768	2665	23.8%	12.4%	7.1%	37.9%	2.8%
42	35 860	17 285	3339	91	1465	1674	19.3%	9.3%	4.7%	43.9%	2.8%
43	28 715	13 072	1946	57	972	877	14.9%	6.8%	3.1%	49.9%	3.0%
44	20 212	8397	970	29	566	350	11.6%	4.8%	1.7%	58.4%	3.1%
45	13 187	5235	419	5	254	145	8.0%	3.2%	1.1%	60.6%	1.2%
46	7480	2740	151	2	90	54	5.5%	2.0%	0.7%	59.6%	1.4%
47	3994	1289	56	1	37	17	4.3%	1.4%	0.4%	66.1%	1.9%
48	1776	522	17	1	11	6	3.3%	1.0%	0.3%	64.7%	6.3%
49	999	301	8	0	5	3	2.7%	0.8%	0.3%	62.5%	0.0%
≥50	705	224	5	0	1	2	2.2%	0.7%	0.3%	20.0%	0.0%

Abbreviation: ET, embryo transfer.

^a^ Multiple pregnancies were defined according to the numbers of gestational sacs in utero.

The treatment outcomes of cycles using frozen‐thawed oocytes based on medical indications are shown in Table 5. There were 136 such FET cycles, among which 38 cycles resulted in a pregnancy (pregnancy rate per FET = 27.9%). The miscarriage rate per pregnancy was 29.0%, resulting in a 15.4% live birth rate per ET.

**TABLE 5:** Treatment outcomes of embryo transfers using frozen‐thawed oocytes based on medical indications in ART clinics in Japan, 2018

Variables	Embryo transfer using frozen‐thawed oocytes
No. of registered cycles	236
No. of ET	136
No. of cycles with pregnancy	38
Pregnancy rate per ET	27.9%
SET cycles	91
Pregnancy following SET cycles	22
Rate of SET cycles	66.9%
Pregnancy rate following SET cycles	24.2%
Miscarriages	11
Miscarriage rate per pregnancy	29.0%
Singleton pregnancies^a^	30
Multiple pregnancies^a^	4
Twin pregnancies^a^	4
Triplet pregnancies^a^	0
Quadruplet pregnancies^a^	0
Multiple pregnancy rate^a^	11.8%
Live births	21
Live birth rate per ET	15.4%
Total number of neonates	23
Singleton live births	19
Twin live births	2
Triplet live births	0
Quadruplet live births	0
Pregnancy outcomes	
Ectopic pregnancies	0
Heterotopic pregnancy	0
Artificial abortions	0
Still births	0
Fetal reduction	0
Unknown cycles for pregnancy outcomes	2

Abbreviations: ET, embryo transfer; SET, single embryo transfer.

^a^ Singleton, twin, triplet and quadruplet pregnancies were defined according to the numbers of gestational sacs in utero.

## 4 DISCUSSION

Using the current Japanese ART registry system for JSOG, there were 454 893 registered ART cycles and 56 979 resultant neonates, The total number of initiated fresh cycles (both IVF and ICSI) increased from the previous year. Freeze‐all cycles predominated, accounting for 47.8% of all initiated fresh cycles. The single ET rate was 82.2% for fresh transfers and 83.4% for frozen‐thawed cycles, which also showed an increasing trend since 2007, reaching a singleton live birth rate of 97% in total. These results represent the latest clinical practice of ART in Japan.

Advanced age for women receiving ART remains one of the most important factors for the increased number of ART cycles in Japan. Thus, in 41.8% of registered cycles, women were in their 40s, similar to the previous year (41.9%), but decreased somewhat from 2015 (43.4%). This might be attributed to the encouragement offered to younger couples having infertility problem to enter ART programs; the Japanese government provides incentives for women under 40s to receive six subsidies for ART to reduce the economic burden, whereas women aged 40‐42 can only receive a subsidy for three attempts. There is currently an upper limit of 73 000 000 JPY per household for the subsidies, but this policy might be changed because of the recent stagnation of the Japanese total fertility rate (TFR). The latest TFR of Japan for 2019 was 1.36,^4^ which had decreased from 1.42 in 2018.^5^ Establishment of effective policies for reversing this low TFR is an urgent problem in Japan's society.

The number of registered ART cycles, both fresh and frozen ET, increased from 2017 to 2018, but the numbers of neonates born in fresh ET cycles have been decreasing since 2016, mostly attributed to the decreased numbers of fresh ET cycles and increased numbers of freeze‐all cycles in Japan. As a result, more than 47% of fresh cycles were freeze‐all in Japan in 2018. This strategy is beneficial for avoiding complications related to ovarian stimulation, such as ovarian hyperstimulation syndrome (OHSS), especially in high‐risk patients such as those with polycystic ovary syndrome or high ovarian reserve.^6^ Supraphysiological hormonal environment in fresh ET cycles might affect endometrial receptivity and implantation rate.^7^ However, clinical evidence on the effect of a freeze‐all strategy for women with regular menstrual cycles is conflicting.^8^, ^9^, ^10^ A recently published meta‐analysis including 11 randomized controlled trials (RCTs) with 5379 patients reported that freeze‐all and subsequent elective FET demonstrated that importantly, neither the live birth rate (relative risk, RR 1.03, 95% confidence interval [CI], 0.91‐1.17) in the subgroup of normal responders nor the cumulative live birth rate in the entire population (RR 1.04, 95% CI, 0.97‐1.11) were significantly different between the two groups.^7^ On the other hand, a more recent multicenter RCT investigating the effect of blastocyst‐stage ET after freeze‐all or fresh ET cycles among 825 ovulatory women from China demonstrated that a freeze‐all strategy with subsequent elective FET achieved a significantly higher live birth rate than did fresh blastocyst ET (RR 1.26, 95% CI, 1.14‐1.41).^8^ The mean age of participants was 28.8 years both for the fresh and frozen ET groups, and the mean number of aspirated oocytes was 14. Thus, we should be cautious when extrapolating these results to the Japanese population. A recent multicenter RCT conducted in Europe investigated the effect of freeze‐all and fresh blastocyst ET strategies with a gonadotropin releasing hormone agonist used to trigger final oocyte maturation among 460 women with regular menstrual cycles (mean age of participants 32.4 years in the freeze‐all group and 32.3 years in fresh ET group) demonstrated that the ongoing pregnancy rate, live birth rate, and obstetric and neonatal complications did not differ between the two groups. The study concluded that their findings warrant caution in the indiscriminate application of a freeze‐all strategy when there is no apparent risk of OHSS.^10^

The strength of the Japanese ART registry is its mandatory reporting system with a high compliance rate, in cooperation with the government subsidy system. Using this system, nearly all participating ART facilities (621 of 622 facilities) have registered cycle‐specific information, so selection bias caused by a lack of participation in the registration system is unlikely. Nevertheless, several limitations exist in the registry. First, it includes only cycle‐specific information, so it is very difficult to identify cycles in the same patient using the registry in its current format. Given recent Japanese ART practice, in which nearly half of all initiated cycles are freeze‐all, widely used indicators such as pregnancy and live birth rates per aspiration cycle will be affected markedly, which could mislead public opinion regarding the quality of treatment as ET was not performed in most included fresh ART cycles.^11^ It has been suggested recently that the cumulative live birth rate per oocyte aspiration is more suitable when reporting the success rate of ART outcomes.^12^, ^13^ Because the Japanese ART registry has asked for frozen cycles to include identification numbers for fresh cycles since 2014, using the information the cumulative live birth rate per aspiration might be informative in the future for Japan as nearly half of all cycles are freeze‐all. Second, the Japanese ART registry includes unfertilized oocyte freezing cycles only for medically indicated cases, such as fertility preservation in cancer patients (Tables 2 and 5); the registry does not include cycles with non‐medical indications: so to speak, “social oocyte freezing.” Because no other registration system currently exists for oocyte freezing, there is no information available for such cycles. Third, the registry includes several patient background factors for each cycle such as body mass index, numbers of previous pregnancies and parity, but the high missed reporting rate for those variables makes it impossible to adjust for them to calculate the success rate for ART in Japan.

In conclusion, our analysis of the ART registry for 2018 demonstrated that the total number of ART cycles increased from the previous year. SET was performed at a rate of more than 82%, resulting in a 97% singleton live birth rate. Although an increasing trend for frozen ET and freeze‐all cycles is evident Japan, further investigation is required to evaluate the effect of the freeze‐all strategy and frozen ET on cumulative live births, particularly with respect to maternal and neonatal safety issues. These data represent the latest clinical practices of ART in Japan. Further improvements in the ART registration system in Japan are important.

## DISCLOSURES

*Conflict of interest*: There is no conflict of interest regarding the publication of this study. *Human rights statement and informed consent*: All procedures were performed in accordance with the ethical standards of the relevant committees on human experimentation (institutional and national) and the Helsinki Declaration of 1964 and its later amendments. *Animal rights*: This report does not contain any studies performed by any of the authors that included animal participants.

## ETHICAL APPROVAL

Not applicable.
